# Iatrogenic ureteric injuries after abdominal surgery: a systematic review and meta‐regression from the European Association of Urology Endourology Section

**DOI:** 10.1111/bju.70275

**Published:** 2026-04-23

**Authors:** Pietro Scilipoti, Nicola Nannola, Federico Zorzi, Carlos Gonzalez Gonzalez, Marie Chicaud, Luigi Candela, Stefano Moretto, Carlotta Nedbal, Tzevat Tefik, Luca Villa, Eugenio Ventimiglia, Andrea Salonia, Alberto Briganti, Francesco Montorsi, Bhaskar K. Somani, Steeve Doizi, Olivier Traxer, Frederic Panthier

**Affiliations:** ^1^ Endolase Lab, GRC20‐Sorbonne Unversity, PIMM‐Arts et Métiers Paris Tech Paris France; ^2^ Service d’Urologie, Tenon Hôpital Paris France; ^3^ Department of Experimental Oncology/Unit of Urology URI, IRCCS Ospedale San Raffaele Milan Italy; ^4^ Vita‐Salute San Raffaele University Milan Italy; ^5^ Polytechnic University Le Marche Ancona Italy; ^6^ IRCSS San Gerardo Monza Italy; ^7^ Endourology Section, European Association of Urology Arnhem The Netherlands; ^8^ Department of Urology, Istanbul Faculty of Medicine Istanbul University Istanbul Turkey; ^9^ Department of Surgical Sciences Uppsala University Uppsala Sweden; ^10^ University Hospital Southampton NHS Foundation Trust Southampton UK

**Keywords:** ureteric injury, iatrogenic injury, endoscopic repair, ureteric reconstruction, meta‐analysis, strictures

## Abstract

**Objective:**

To systematically evaluate management strategies and associated outcomes of iatrogenic ureteric injuries, focusing on clinical success, renal unit loss, and the need for subsequent endoscopic or reconstructive procedures.

**Patients and Methods:**

A pre‐registered protocol (CRD420251184018) guided a comprehensive search of PubMed and EMBASE databases. Observational studies reporting outcomes of ≥20 patients treated for iatrogenic ureteric injury with complete data on follow‐up were included. Risk of bias was assessed using Risk Of Bias In Non‐randomised Studies of Interventions (ROBINS‐I). Random‐effects meta‐analysis with PLOGIT transformation was applied, stratifying results by treatment type and timing. Meta‐regression examined the association of treatment modality with outcomes adjusting for the timing of reconstruction.

**Results:**

A total of 30 studies were included (1517 patients), all retrospective and characterised by substantial heterogeneity in definitions and follow‐up practices. ROBINS‐I indicated serious risk of bias in most domains. The pooled clinical success rate was 87% (95% confidence interval [CI] 80–91%), with high heterogeneity *I* ^2^ = 76.0% due to different follow‐up protocols, success outcomes, type and timing of management. Studies dominated by reconstructive procedures (15 studies) had higher success (91%, 95% CI 84–95%), than those primarily using endoscopic techniques (six studies; 66%, 95% CI 58–73%). Renal unit loss occurred in 2.3% (95% CI 1.5–3.6%). No differences in loss of renal unit were found. Additional endoscopic interventions were required in 3% (95% CI 2–4%) and further reconstructive surgery in 5% (95% CI 3–9%). Meta‐regression showed endoscopic index management was associated with lower odds of success (odds ratio [OR] 0.14, 95% CI 0.05–0.40) and higher need for further reconstruction (OR 5.64, 95% CI 1.61–19.8).

**Conclusions:**

Across retrospective and heterogeneous studies, reconstructive‐dominant management of iatrogenic ureteric injuries was associated with higher clinical success compared with endoscopic‐dominant approaches, while renal unit loss remained uncommon. Endoscopic management was feasible in selected cases but was more frequently followed by additional endoscopic or reconstructive interventions.

**Abbreviations**
IUI: iatrogenic ureteric injury
OR: odds ratio
PRISMA: Preferred Reporting Items for Systematic Reviews and Meta‐Analyses
ROBINS‐I: Risk Of Bias In Non‐randomised Studies of Interventions

## Introduction

Iatrogenic ureteric injuries (IUIs) represent uncommon but potentially devastating complications of abdominopelvic and endoscopic surgery. Their incidence is estimated at 0.3–1.5% in major pelvic operations, with gynaecological, colorectal, and urological procedures accounting for most cases [1]. Although infrequent, these injuries can lead to severe morbidity, including urinary leakage, sepsis, loss of renal function, and chronic ureteric stricture, particularly when diagnosis is delayed. Early recognition and appropriate management are therefore critical to prevent long‐term sequelae and preserve renal integrity [2].

Traditionally, open or laparoscopic reconstructive techniques such as ureteroureterostomy, ureteroneocystostomy, and Boari flap repair have been considered the ‘gold standard’ for definitive treatment according to the location of the lesion. However, these procedures often require specialized expertise, are associated with considerable operative morbidity, and may not be immediately feasible in patients with complex postoperative or inflammatory anatomy [3]. In recent years, advances in minimally invasive and endourological approaches have provided less‐invasive alternatives for both temporary and definitive management [4]. Techniques including retrograde ureteric stenting, percutaneous nephrostomy, balloon dilatation, and endoureterotomy have demonstrated promising results in restoring ureteric patency, controlling leakage, and avoiding major reconstruction in selected patients [3, 5].

Despite these advances, current evidence remains highly heterogeneous. Published studies are predominantly retrospective, include small and diverse patient populations, and differ widely in terms of injury mechanism, timing of diagnosis, and definitions of treatment success. Current guidelines provide guidance regarding the optimal management of IUIs; however, there is lack of solid benchmarks and outcomes of these management recommendations that summarise literature based on success rate, ureteric patency, renal function preservation, and the need for delayed reconstruction remain limited.

## Patients and Methods

### Search Strategy

A systematic search of PubMed, EMBASE, and the Cochrane Library was conducted in accordance with Preferred Reporting Items for Systematic Reviews and Meta‐Analyses (PRISMA) guidelines (International Prospective Register of Systematic Reviews [PROSPERO] number: CRD420251184018; Files S1 and S2). Search strategies combined controlled vocabulary and free‐text terms relating to ‘ureteral injury’, ‘iatrogenic complications’, ‘endoscopic management’, and ‘ureteral reconstruction’. References of eligible articles and prior reviews were also screened for additional studies.

### Eligibility Criteria

Studies were eligible if they met all of the following inclusion criteria: (i) adult patients (aged ≥18 years) with IUI occurring during or after abdominopelvic surgery; (ii) management with reconstructive and/or endourological techniques, including ureteric stenting, percutaneous nephrostomy, ureteroscopy‐based realignment, balloon dilatation, or endoureterotomy; (iii) original data including ≥20 treated patients; (iv) randomized, prospective, or retrospective observational studies.

Studies were excluded if they: (i) reported exclusively non‐IUIs (e.g., blunt or penetrating trauma); (ii) focused solely on prophylactic ureteric stenting without documented ureteric injury; (iii) were case reports (<20 patients), reviews, editorials, or expert opinions; or (iv) were not published in English. Studies including ureteric injuries not related to abdominopelvic surgery were included only when these represented ≤20% of the overall cohort.

**Fig. 1:**
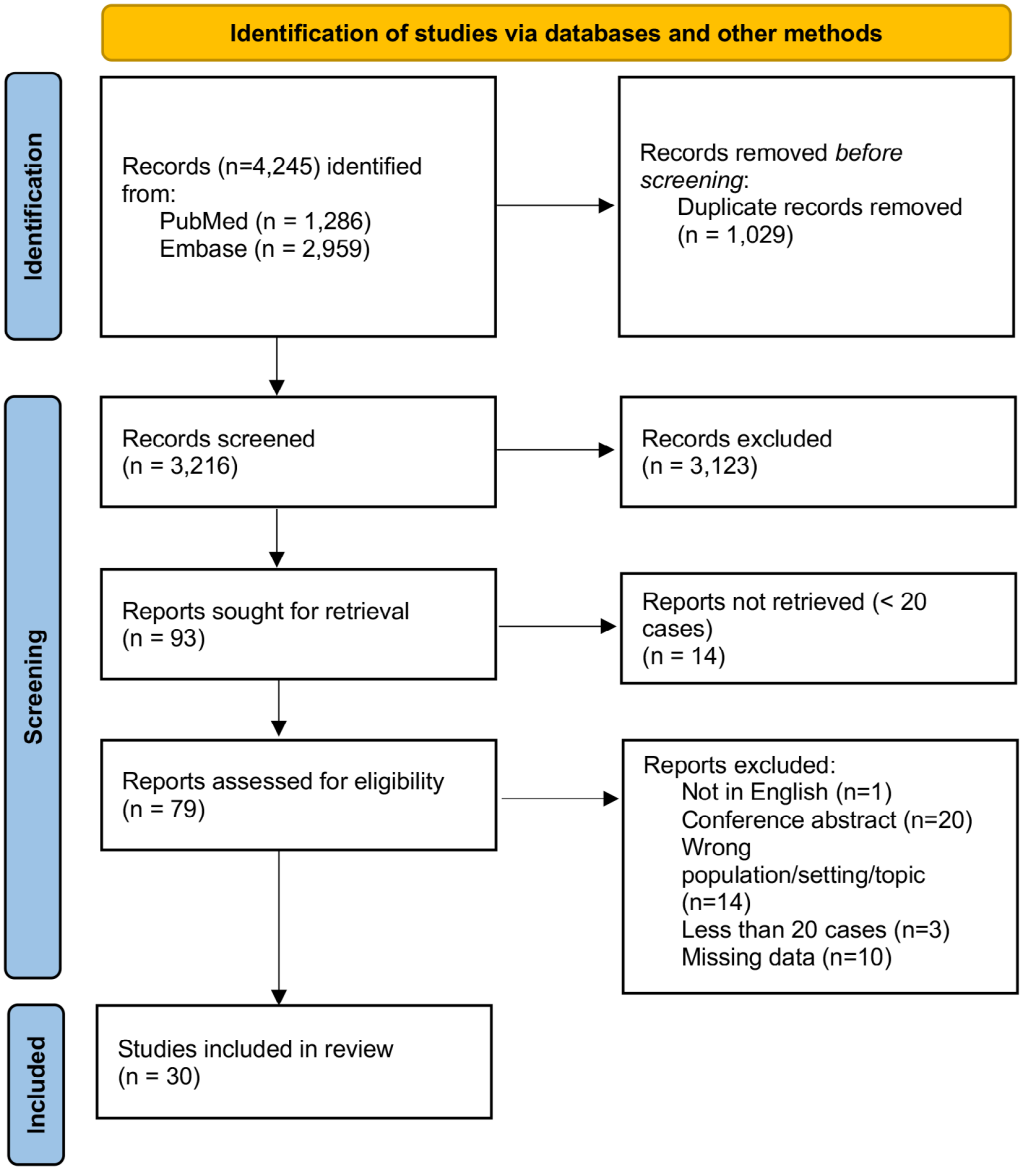
The PRISMA flowchart of study selection.

Study selection was performed independently by two reviewers (N.N., C.G.), with disagreements adjudicated by a third reviewer (P.S.). The study selection process and reasons for exclusion at the full‐text stage are reported in the PRISMA flow diagram (Fig. 1).

### Data Extraction

Data were extracted independently by two reviewers using a standardised form. The following information was collected: study characteristics: first author, year, country, design, sample size, recruitment period; patient demographics: age, sex, primary surgical procedure, indication (benign vs malignant); injury characteristics: side, location, mechanism (ligation, transection, thermal, avulsion, etc.), timing of diagnosis; intervention details: type and timing of invasive/endourological management, number of procedures, adjunctive measures, and need for subsequent reconstruction.

The primary outcome was clinical success as defined by each study. Secondary outcomes were loss of renal unit, need for further endoscopic procedures, and need for additional reconstructive surgery.

### Definition of Treatment Modality and Timing

To standardise comparisons across heterogeneous studies, management strategies were categorised based on explicit procedural descriptions in the source articles.

#### Treatment Modality

- Endoscopic management: retrograde or antegrade minimally invasive techniques including ureteric stenting, percutaneous nephrostomy with internal drainage, balloon dilatation, cold‐knife incision, or laser endoureterotomy. These procedures did not involve open or robot‐assisted reconstruction.
- Reconstructive management: open, laparoscopic, or robot‐assisted reconstructive procedures aimed at restoring ureteric continuity or function, including ureteroureterostomy, ureteroneocystostomy (with/without psoas hitch or Boari flap), transureteroureterostomy, ileal ureter substitution, and other less commonly reported techniques (e.g., renal autotransplantation). These constituted definitive repairs rather than endoscopic temporising measures.
- Mixed management: studies reporting treatment outcomes for heterogeneous cohorts combining endoscopic and reconstructive procedures without stratified results, or staged hybrid approaches.

A cut‐off of 80% was used to define one cohort or the other. When the difference was <80% the cohort was defined as mixed.

#### Timing of Treatment

Definitions were harmonised according to conventions used in the majority of included studies. A more granular temporal stratification (e.g., immediate, early, and delayed based on fixed day thresholds) was not feasible due to heterogeneous and inconsistently reported timing definitions across studies, often lacking uniform cut‐offs or patient‐level data. Accordingly, management timing was categorised as follows:

- Immediate intervention: treatment performed during the index surgery.
- Delayed intervention: treatment not performed during the surgery when the damage was performed. This category included repairs labelled as ‘delayed’, ‘late’, or ‘secondary’.
- Mixed timing: studies that included both immediate and delayed treatments.

A cut‐off of 80% was used to define if a cohort was mostly delayed or immediate. These standardised definitions were used consistently across subgroup analyses and meta‐regression.

### Risk of Bias Assessment

Two reviewers independently assessed the methodological quality of each study. For randomised controlled trials, the Cochrane Risk of Bias 2 (RoB 2) tool was used. For non‐randomised or observational studies, the Risk Of Bias In Non‐randomised Studies of Interventions (ROBINS‐I) tool was applied. Each domain (confounding, selection, classification, deviations, missing data, outcome measurement, and reporting bias) was evaluated, and results were presented graphically and in tabular form.

### Data Synthesis and Statistical Analysis

Random‐effects meta‐analysis using a PLOGIT transformation was applied to stabilise variances for proportion outcomes, particularly those involving rare events. Heterogeneity across studies was quantified using *I* ^2^ and *τ* ^2^. Subgroup meta‐analyses were conducted stratifying results according to treatment modality and timing since recognition and treatment.

An overall assessment of these outcomes in study without serious risk of bias was performed.

### Meta‐Regression

A meta‐regression model was performed to evaluate the association between the type of primary management of ureteric injury and each of the included outcomes adjusting for timing to surgery (immediate vs delayed).

Statistical significance was considered at *P* < 0.05. For all statistical analyses, R software environment for statistical computing and graphics was used (version 4.5.1; http://www.r‐project.org/).

## Results

### Characteristics of Included Studies

A total of 30 retrospective observational studies comprising 1517 patients were included, with no prospective or randomised designs identified. Definitions of clinical success varied widely, from symptom‐based assessments to strict radiological documentation of ureteric patency and were often incompletely reported. Follow‐up intervals ranged from a few months to over a decade, reflecting major inconsistencies in postoperative monitoring. Imaging protocols were neither standardised nor systematically applied. Many studies lacked details on timing of intervention, severity of injury, or functional renal assessment, limiting comparability across cohorts. Several series combined endoscopic and reconstructive treatments without stratifying outcomes, further contributing to heterogeneity. No study performed routine endoscopic upper tract reassessment before removal of urinary drainage. Overall, available evidence derives from small, heterogeneous cohorts with substantial methodological limitations (Table 1 [1–31]).

**Table 1:** Baseline characteristics of the 30 studies included in the systematic review.

Study	Study period	Country	Age, years	Design	Sample size	Main index surgery of injury	Main mechanism of injury
Ihse et al. (1975) [6]	1956–1972	Sweden	NR	Retro	42 pts (4 bilat)	Gyn 55%; urological 31%; GI 12%; retroperitoneal tumour 2%	Transection 10; ligation 3; ischaemic stricture 22
Witters et al. (1986) [22]	1980–1984	Belgium	24–74	Retro	26 pts (2 bilat)	Gyn procedures main group (18 injuries); others: vascular, colorectal, appendectomy, APR, stone manipulation	Ligation/partial transfixation 13; complete ligation 7; complete/partial transection 10; necrosis 2; prolapse 1
Lask et al. (1995) [7]	1979–1992	Israel	Mean 59	Retro	44 pts	Gyn surgery 82%; colorectal/abdominal 14%; urologic 5%	NR
Giberti et al. (1996) [8]	1982–1994	Italy	Mean 51	Retro	63 pts (9 bilat)	Almost all gyn: abdominal, radical or vaginal hyst; Burch; C‐section; ovariectomy	NR
Kostakopoulos et al. (1998) [9]	1987–1995	Greece	Mean 53	Retro	40 pts (6 bilat)	Gyn surgery (abdominal or vaginal hyst)	NR
Brandt et al. (2001) [10]	1981–1999	Brazil	Mean 36.5	Retro	47 pts	TAH 81%; vaginal hyst 9%; salpingo‐oophorectomy 4%; urethropexy 6%	NR
Sakellariou et al. (2002) [15]	1987–1996	Greece	NR	Retro	76 pts (12 bilat)	Mainly hyst (abdominal, vaginal, malignancy), pelvic clearance, ovarian tumour surgery, intrapartum hyst	Ligation 38; partial transection 8; complete transection 10; wall injury 8; ischaemic necrosis ± UVF 11
Ku et al. (2003) [23]	1985–1999	S. Korea	51 (24–76)	Retro	30 pts (3 bilat)	Hyst 53%; abdominal hyst 27%; C‐section 17%; oophorectomy 3%	NR
Yuvaraja et al. (2003) [19]	1982–2001	India	Mean 47	Retro	34 pts (2 bilat)	Open radical hyst 100%	Partial transection 8; complete 9; crushing 2; adventitial injury 3; ligation 2; segment resection 3
El‐Tabey et al. (2006) [24]	1985–2003	Egypt	Mean 34	Retro	120 pts	Obstetric–gyn: hyst 56%; C‐section 24%; obstructed labour 20%	NR
Singh et al. (2010) [25]	2004–2008	India	Mean 32	Retro	24 pts (6 bilat)	Abdominal hyst (benign/malignant), C‐section, tubo‐ovarian mass surgery, cystocele repair	NR
Koukouras et al. (2010) [4]	NR	Greece	Mean 59	Retro	24 pts (1 bilat)	Gyn 10; colorectal 7; urological 3; other 4; PCNL/pyeloplasty 2	Ligation/suture entrapment 18; disruption/laceration/avulsion 7 (coagulation often co‐existing)
Abraham et al. (2011) [20]	2006–2010	India	Mean 36	Retro	36 pts (1 bilat)	Gyn 33 hyst + 1 mesh; colorectal 1; urological 1	NR
Park et al. (2012) [1]	2006–2011	S. Korea	Mean 50	Retro	35 pts (3 bilat)	Mix of gyn and colorectal/urological procedures (not detailed)	NR
Zilberman et al. (2015) [21]	2001–2013	Israel	Median 44	Retro	29 pts	Mainly gyn lap/open procedures, C‐section, adhesiolysis, hysteroscopy; few colorectal	NR
El Abd et al. (2015) [11]	1986–2014	Egypt	Median 27	Retro	98 pts	Mostly hyst (open/lap), C‐section, adhesiolysis, oophorectomy, hysteroscopy, colon LAR	NR
Chung et al. (2016) [17]	1999–2011	UK	Mean 46	Retro	25 pts	Gyn 76%; general surgery 12%; urological 8%; other 4%	Blade transection/laceration 15; laser 6; scope‐related 4
Jiao et al. (2017) [14]	2009–2016	China	Mean 51	Retro	40 pts (2 bilat)	Gyn; retroperitoneal tumour; urological; other	Complete transection 26; partial injury 9; combined 5
Wang et al. (2017) [26]	2010–2016	China	NR	Retro	60 pts	Gyn surgery 100%	UVF 17; obstruction/stenosis 28; transection 15
Tseng et al. (2018) [12]	2007–2016	Taiwan	53.6 (IQR 46–63)	Retro	70 pts (2 bilat)	Gyn 63%; colorectal 29%; urological 9%	Complete transection, ligation, or segment loss (not further split)
Yapanoglu et al. (2018) [13]	2005–2015	Turkey	Mean 43	Retro	30 pts	Oncological gyn 67%; C‐section 33%	Complete transection 10; incomplete 9; UVF 7; ligation 4
Lim et al. (2018) [18]	2005–2017	S. Korea	Mean ~ 45	Retro	102 pts	Mixed open/lap hyst, myomectomy, oophorectomy, radical vaginal hyst	N/A (all strictures after prior repair)
Aguilera et al. (2019) [2]	2004–2016	Spain	Mean: 49	Retro	84 pts	Gyn procedure: 76%; General surgery: 24%	NR
Shchukin et al. (2019) [27]	NR	Ukraine	Mean 46.8	Retro	73 pts (6 bilat)	Obstetric–gyn 86%; URS 4%; rectal surgery 7%; aorto‐femoral bypass 3%	NR
Ambani et al. (2019) [28]	2012–2018	USA	NR	Retro	67 pts	Benign gyn 42%; colorectal 31%; gyn oncology 16%; other (vascular/spine/prostatectomy) 10%	NR
Kim et al. (2021) [31]	2007–2016	S. Korea	Mean 49.6	Retro	61 pts	Lap hyst (benign) 43%; lap Hyst 18%; lap/open colorectal 21%; URS 7%; C‐section 5%; other lap procedures	Incomplete transection 15; complete 12; obstruction/inflammation 9; ligation 8; thermal 7; avulsion 4; unknown 6
Cebeci (2022) [16]	2011–2018	Turkey	Mean 52.6	Retro	27 pts	Lap hyst 70%; open hyst 30%	Thermal injury 20; transection 7
Rahoui et al. (2022) [29]	2005–2020	Tunisia	Mean 44	Retro	32 pts	Open hyst 44%; lap hyst 28%; C‐section 19%; prolapse repair 9%	NR
Grimes et al. (2022) [30]	2010–2018	USA	Mean 54	Retro	47 pts (8 bilat)	Surgical 58%; radiotherapy strictures 28%; external trauma 4%; endoscopic stone 4%; other 6%	NR
Kumar et al. (2025) [5]	2019–2024	India	Mean 46	Retro	31 pts	Post‐hyst 77%; pelvic malignancy surgery 10%; radiotherapy‐related 13%	N/A (all UVFs)

APR, abdominoperineal resection; bilat, bilateral; C‐section, caesarean section; GI, gastrointestinal; gyn, gynaecological; hyst, hysterectomy; IQR, interquartile range; lap, laparoscopic; LAR, low anterior resection; NR, not reported; PCNL, percutaneous nephrolithotomy; pts, patients; Retro, retrospective; TAH, total abdominal hysterectomy; URS, ureteroscopy; UVF, ureterovaginal fistula.

### Type of Ureteric Injury and Index Surgical Procedures

Ureteric injuries originated from a broad spectrum of abdominopelvic operations, though gynaecological surgery consistently accounted for most events across all decades. Early studies such as Ihse et al. [6] (1975) reported that over half of injuries occurred during hysterectomy‐based procedures, a pattern reproduced in later cohorts by Lask et al. [7], Giberti et al. [8], Kostakopoulos et al. [9], and Brandt et al. [10], where gynaecological operations represented more than 70–80% of index surgeries. This trend persisted in contemporary series, including El Abd et al. [11] (2015), Tseng et al. [12] (2018), Yapanoglu et al. [13] (2018), and Aguilera et al. [2] (2019), where 60–80% of injuries followed open or laparoscopic hysterectomy. Other surgery types involved colon and vascular surgery [4, 14].

The mechanisms of injury reflected the surgical approach: ligation and partial or complete transection were dominant in open and laparoscopic pelvic surgery, whereas thermal damage, scope‐related trauma, and ischaemic strictures were more commonly associated with minimally invasive techniques [6, 15, 16, 17, 18]. Reconstructive surgery, primarily ureteroneocystostomy, end‐to‐end anastomosis, psoas hitch, or Boari flap, was the predominant initial management strategy, achieving high success rates (~85–100%) across most cohorts, including Giberti et al. [8] (1996), Brandt et al. [10] (2001), Yuvaraja et al. [19] (2003), Abraham et al. [20] (2011), and Aguilera et al. [2] (2019) .

Endoscopic management was less frequently used as definitive therapy but displayed substantial technical variability, ranging from retrograde stenting and nephrostomy to balloon dilatation and endoureterotomy. Success varied widely (~57–90%) and depended heavily on injury severity and timing, with favourable outcomes in partial transections (e.g., 100% in Yapanoglu et al., 2018 [13]; 88% in Giberti et al., 1996 [8]) but poorer results in complete transections or thermal injuries, which often required secondary reconstruction. Balloon dilatation was mainly used for early strictures, while staged nephrostomy with antegrade stenting was frequently applied in delayed or complex cases [18, 21]. Importantly, no study reported systematic endoscopic follow‐up to confirm healing, limiting the reliability of ‘success’ definitions across the literature.

Baseline characteristics of the other studies included in the review are listed in Table 1.

### Risk of Bias

**Fig. 2:**
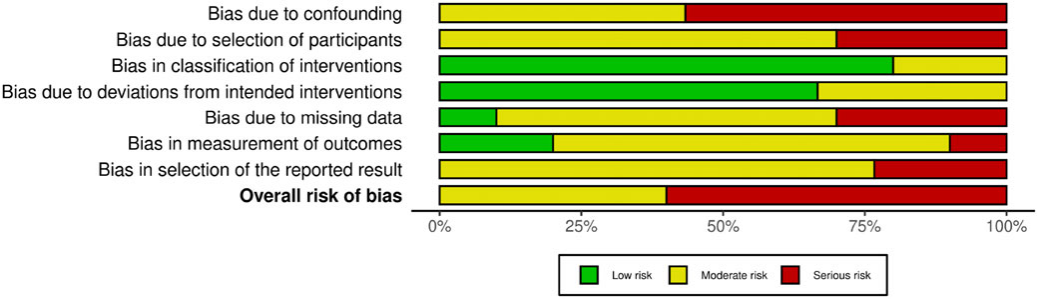
Domain‐level risk of bias across all included observational studies assessed using the ROBINS‐I framework.

Overall risk of bias was substantial. Most included studies were judged to have a serious risk of bias in at least one ROBINS‐I domain (Fig. S1). Common limitations included inadequate control for baseline confounding and the absence of pre‐specified analytical plans (Fig. 2).

### Clinical Success Rate

Across all management modalities, the pooled clinical success rate was high, although heterogeneity remained considerable. All 30 studies reported outcomes of clinical success (Table S1).

**Fig. 3:**
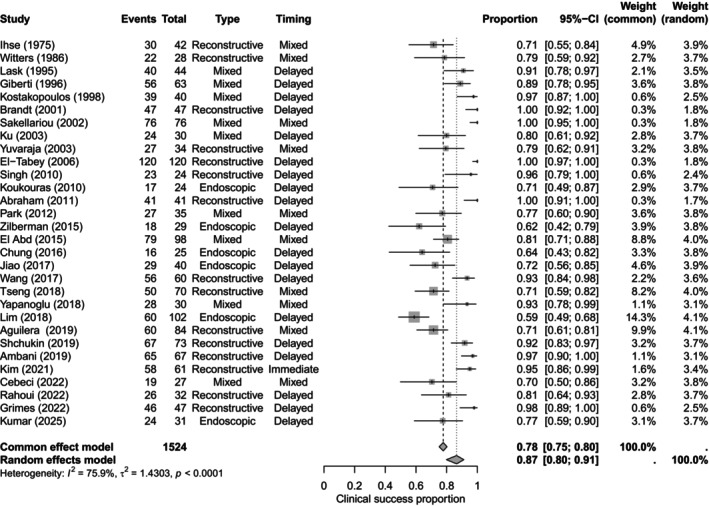
Pooled proportion of clinical success rate across included studies.

The overall pooled rate of success was 87% (95% CI 80–91%) with high heterogeneity (*I* ^2^ = 76%, *P* < 0.001) (Fig. 3). When considering only studies without high risk of bias the results remained consistent 85% (95% CI 73–92%, *I* ^2^ = 74%, *P* < 0.001).

When stratifying according to procedure, studies with a majority of reconstructive procedure (15 studies) as the primary management had a pooled rate of clinical success of 91% (95% CI 83–95%, *I* ^2^ = 77%, *P* < 0.001), on the contrary the pooled rate of studies with a majority of endoscopic correction procedures (six studies) was 66% (95% CI 58–73%, *I* ^2^ = 3.7%) (Table 2).

**Table 2:** Pooled estimation of success rates according to subgroups of treatment and timing of treatment.

All studies (*N* = 30)		
Type	Success rate, % (95% CI)*	Heterogeneity
Primary management		
Endoscopic (*n* = 6)	66 (58–73)	*I* ^2^ = 3.7%, *P* = 0.4
Mixed (*n* = 9)	88 (77–94)	*I* ^2^ = 60%, *P* = 0.010
Reconstructive (*n* = 15)	91 (83–95)	*I* ^2^ = 77%, *P* < 0.001
Timing of treatment		
Immediate/mixed (*n* = 11)	82 (71–90)	*I* ^2^ = 60%, *P* = 0.006
Delayed (*n* = 19)	89 (81–94)	*I* ^2^ = 81%, *P* < 0.001
Without high risk of bias* (*n* = 12)		
Primary management		
Endoscopic (*n* = 4)	69 (58–77)	*I* ^2^ = 0%, *P* = 0.6
Mixed (*n* = 3)	85 (70–93)	*I* ^2^ = 67%, *P* = 0.046
Reconstructive (*n* = 5)	94 (81–98)	*I* ^2^ = 83%, *P* < 0.001
Timing of treatment		
Immediate/mixed (*n* = 3)	82 (55–94)	*I* ^2^ = 81%, *P* = 0.004
Delayed (*n* = 9)	86 (72–94)	*I* ^2^ = 73%, *P* < 0.001

A cut‐off of 80% was used to define a study as endoscopic vs reconstructive vs mixed. The same cut‐off was used to define the timing where immediate repair was defined as treatment intraoperatively.

* Random effect models.

Clinical success according to different timing of intervention and recognition varied also. When pooling results of studies with delayed recognition the success rate was 89% (95% CI 81–94%), while the success rate in mixed cases and one immediate study was similar 82% (95% CI 71–90) (Table 2).

### Renal Unit Loss Rate

Across 30 studies reporting this endpoint, the overall renal unit loss rate was low, with a pooled proportion of 2.3% (95% CI 1.5–3.6%), with low heterogeneity (Fig. S2).

### Further Necessity of Endoscopic Treatment

The need for additional endoscopic interventions varied widely across studies, with a pooled proportion of ~3% (95% CI 2–4%) with moderate heterogeneity (Fig. S3).

### Further Necessity of Reconstructive Treatment

Reconstructive re‐intervention occurred in 5% (95% CI 3–9%) of patients overall, with high variability driven by injury severity (Fig. S4).

### Meta‐Regression Analysis

The endoscopic management cohort showed an association with lower odds of clinical success (odds ratio [OR] 0.13, 95% CI 0.04–0.39; *P* < 0.001) and higher risk of necessity of further reconstructive surgery (OR 6.39, 95% CI 1.69–24.1, *P* = 0.006) (Table 3).

**Table 3:** Meta‐regression analysis according to type of procedure in studies without serious risk of bias.

Outcome	Characteristics	OR (95% CI)	*P*
Clinical success	Procedure type*		
	Reconstructive	Ref	
	Mixed cohort	0.94 (0.37–2.41)	0.9
	Endoscopic	0.13 (0.04–0.39)	**<0.001**
Loss of renal unit	Reconstructive	Ref	
	Mixed cohort	0.77 (0.28–2.12)	0.6
	Endoscopic	0.50 (0.11–2.27)	0.4
Further endoscopic surgery	Reconstructive	Ref	
	Mixed cohort	2.88 (0.92–8.99)	0.069
	Endoscopic	2.86 (0.72–11.4)	0.14
Further reconstructive surgery	Reconstructive	Ref	
	Mixed cohort	2.12 (0.63–7.13)	0.2
	Endoscopic	6.39 (1.69–24.1)	**0.006**

The models implemented were adjusted for the aggregated timing of treatment (Immediate vs Mixed vs Delayed).

Bold values statistically significant at *P* < 0.05.

* A cut‐off of 80% was used to define a cohort with a different type of procedure. Studies defined as mixed were those with balanced distribution of endoscopic and reconstructive only procedures.

## Discussion

This systematic review offers the most comprehensive synthesis to date of minimally invasive and reconstructive strategies for managing IUIs across abdominopelvic surgery. By pooling data from more than 1517 patients, this review highlights recurrent clinical patterns while underscoring substantial evidence gaps that limit confident guideline development. However, interpretation of the findings must be carefully framed within the substantial methodological limitations of the included literature. All available data derive from retrospective studies characterised by marked heterogeneity in injury mechanisms, timing of diagnosis, treatment selection, definitions of clinical success, and follow‐up strategies. As such, the results of this review should be considered primarily descriptive and hypothesis‐generating rather than comparative or causal.

Across studies, overall clinical success exceeded 80%, yet this aggregate figure conceals marked variability driven by differences in injury mechanism, diagnostic timing, and the nature of the primary intervention. Reconstructive surgery (e.g., open, laparoscopic, or robot‐assisted) consistently achieved the highest and most durable success, with a pooled success rate of 94% among studies without serious risk [32]. These repairs aim to restore ureteric continuity and vascularity, and their outcomes remained robust even when performed after delayed recognition. Conversely, endoscopic management demonstrated broader variability, reflecting its sensitivity to injury grade, tissue viability, timing, and the substantial heterogeneity of techniques used across studies [18, 21].

Timing of diagnosis emerged as a central dimension of this review. Immediate intervention was uncommon and only one study met the prespecified 80% intraoperative‐recognition threshold, as most injuries were discovered postoperatively. Importantly, the heterogeneity and imprecision in reporting time to diagnosis and repair across studies precluded a more granular stratification of timing (e.g., early vs late based on fixed temporal cut‐offs), limiting direct comparison of narrower time windows. Despite guideline recommendations and multiple series suggesting superior recovery after immediate repair, intraoperative recognition remains challenging, especially in complex procedures, and requires notable surgical expertise [2, 3, 33, 34]. Preoperative stenting may facilitate intraoperative identification in selected high‐risk cases [3]. Owing to procedural heterogeneity, no consistent difference emerged between cohorts with high vs low prevalence of immediate repair. Although some studies reported greater success with immediate intervention [1, 16, 22], others showed no clear advantage [10, 11, 15]. This likely reflects that success depends not solely on timing but on the appropriateness and precision of the selected technique.

Renal unit loss was infrequent, although significant (~2–3% pooled). This low nephrectomy rate should be interpreted cautiously, as most studies lacked standardised follow‐up imaging and did not employ objective functional assessments such as renography or differential renal function. Notably, no study implemented rigorous endoscopic surveillance after primary repair, raising the possibility that delayed strictures remained undetected unless symptomatic. This highlights a broader limitation: clinical ‘success’ was often judged by symptom resolution or discretionary imaging rather than pre‐defined follow‐up protocols.

The need for secondary procedures provides an indirect measure of primary treatment durability. Endoscopic management was associated with a markedly higher likelihood of requiring delayed reconstructive surgery, approximately a fivefold increase compared with initial reconstructive repair. However, this association must be interpreted with caution. Endoscopic management was frequently employed as an initial or stepwise strategy in selected, anatomically favourable injuries, whereas more extensive, ischaemic, or complex lesions were preferentially managed with primary reconstruction. Conversely, it is also plausible that easier cases were selected for endoscopic management, making interpretation of outcome differences even more uncertain. Neither indication bias nor reverse selection bias can be reliably disentangled using the available data.

Given the lack of conclusive data from the literature, we sought to propose the following considerations, which are presented explicitly as expert opinion and hypothesis‐generating concepts, rather than conclusions supported by the present meta‐analysis. When the ureteric lesion is identified intraoperatively, immediate reconstructive repair should be favoured whenever feasible, as this approach consistently achieves the highest and most durable success rates across studies. Conversely, when the injury is not recognised at the time of surgery, early urinary diversion remains essential to control leakage, prevent infection, and preserve renal function. In this setting, prompt placement of a JJ ureteric stent, with or without percutaneous nephrostomy depending on the degree of obstruction or urinoma, should be considered as a temporising but critical first step to promote ureteric healing and allow tissue stabilisation. Importantly, endoscopic management should not be viewed as a definitive solution without structured reassessment. Our review highlights that most endoscopic series lacked standardised follow‐up and objective confirmation of ureteric healing. We propose that the optimal endoscopic pathway should systematically include scheduled stent removal followed by endoscopic reassessment of the ureteric segment to evaluate residual stenosis, ischaemia, or fibrosis (using rigid or flexible ureteroscopy). Only patients demonstrating adequate ureteric patency on objective endoscopic and imaging evaluation should be classified as successfully treated endoscopically. In this context, functional assessment should complement anatomical evaluation, as a scarred ureter may remain functionally obstructive despite apparent luminal continuity; accordingly, MAG3 renal scintigraphy in conjunction with estimated GFR assessment should be considered to proper drainage and differential renal function.

The currently available evidence does not allow clear identification of which patients benefit most from each endoscopic modality, the optimal timing of these interventions relative to injury recognition and tissue stabilisation, or the appropriate sequencing of techniques. Across studies, these approaches are inconsistently described, applied with substantial technical variability, and rarely supported by standardised definitions of success or mandatory post‐treatment endoscopic confirmation.

This study has limitations. Substantial heterogeneity across outcomes warrants emphasis. Definitions of success, timing thresholds, imaging strategies, injury classifications, and follow‐up duration varied widely, complicating cross‐study comparisons. Few studies stratified patients by mechanism of injury (ligation, transection, thermal injury), though these distinctions critically influence the feasibility of minimally invasive repair. Thermal injuries, for example, may initially appear minor but progress to full‐thickness necrosis, making early endoscopic success misleading without structured reassessment. Similarly, reporting of proximal vs distal injuries or partial vs complete transections was inconsistent across many cohorts [1, 2, 7, 8, 9, 10, 11, 20, 23, 24, 25, 28]. Methodological quality was another major concern: all studies were retrospective, and ROBINS‐I assessments revealed moderate to serious risk of bias in most domains. Confounding was rarely addressed, baseline characteristics were often incomplete, and selection bias was common, reflecting variable referral patterns and operator expertise. Additionally, the definition of success was heterogeneous and provided exclusively as a binary outcome without time‐dependent descriptions. These limitations weaken causal inference regarding comparative effectiveness. For example, lower success rates of delayed endoscopic management may reflect the selection of more complex injuries for such attempts rather than true inferiority of the technique. Without prospective designs and standardised grading, these confounders cannot be adequately resolved.

In addition, some included series contained a small proportion of ureteric injuries not related to abdominopelvic surgery. Although this proportion was limited to ≤20% to preserve the feasibility of quantitative synthesis, residual heterogeneity across studies cannot be entirely excluded and may have influenced outcome estimates.

Despite these limitations, this systematic review provides several actionable insights. Endoscopic management remains a reasonable option, achieving nearly 70% success in many series. Reconstructive repair maintains high success across time points and remains the most reliable definitive approach, particularly for high‐grade or delayed injuries [3]. Given the heterogeneity of treatment selection across studies, management should be tailored to individual injury characteristics and patient factors. Finally, the review highlights the urgent need for prospective, standardised research incorporating unified injury classifications, precise outcome definitions, and standardised criteria regarding the timing of repair, in order to support clearer and evidence‐based recommendations for future practice. Structured imaging and endoscopic follow‐up protocols, as well as comparative effectiveness analyses stratified by mechanism and timing of injury, are also warranted. Multicentre registries or trials would meaningfully advance the field beyond the limitations of small retrospective series.

In conclusion, this systematic review demonstrates that the existing literature on IUIs is limited by heterogeneity, bias, and lack of prospective data. Reconstructive repair is associated with high success rates across studies, while endoscopic management may be feasible in selected cases but shows more variable outcomes. Given the quality of the available evidence, management decisions should be individualised, taking into account injury characteristics, timing of diagnosis, and surgical expertise. Well‐designed prospective studies with standardised definitions, uniform reporting, and structured follow‐up are urgently needed to refine treatment selection and to validate management paradigms suggested by retrospective experience.

## Disclosure of Interests

Olivier Traxer has declared as a consultant for Karl Storz, Coloplast, IPG Photonics, Quanta System, and Rocamed. Frédéric Panthier has declared as consultant for Dornier MedTech. Steeve Doizi has declared consultant for Dornier MedTech and Coloplast. Bhaskar Somani has declared consultant for Boston Scientific, Coloplast, Pusen, GSK and NovoNordisk. All other authors have no conflicts of interest related to the present study.

## Supporting information


**Fig. S1.** Domain‐level risk‐of‐bias assessment for all included observational studies using the ROBINS‐I framework. Each row represents a study, and each column corresponds to one of the seven ROBINS‐I domains (D1–D7).
**Fig. S2.** Pooled proportion of renal unit loss across included studies (common‐effect and random‐effects models). Individual study estimates are displayed with 95% CIs, stratified by type of intervention and timing of diagnosis.
**Fig. S3.** Meta‐analysis of the proportion of patients requiring further endoscopic intervention following IUI. Study‐specific proportions and 95% CIs are shown alongside pooled estimates under common‐effect and random‐effects models.
**Fig. S4.** Pooled proportion of patients requiring additional reconstructive surgery after ureteric injury. Individual study estimates with 95% CIs are reported together with overall pooled effects (common‐effect and random‐effects models).
**Table S1.** Overview of management strategies and corresponding clinical success rates across studies included in the systematic review.

**File S1.** The PRISMA 2020 checklist.

**File S2.** Search strategies.
