# Supplementary material for: Iatrogenic ureteric injuries after abdominal surgery: a systematic review and meta‐regression from the European Association of Urology Endourology Section
Source: BJU Int. 2026 Apr 23;138(1):45–56. doi: 10.1111/bju.70275 (PMC13244928; doi:10.1111/bju.70275)
Supplement: Supplementary file 1 — Fig. S1. Domain‐level risk‐of‐bias assessment for all included observational studies using the ROBINS‐I framework. Each row represents a study, and each column corresponds to one of the seven ROBINS‐I domains (D1–D7). Fig. S2. Pooled proportion of renal unit loss across included studies (common‐effect and random‐effects models). Individual study estimates are displayed with 95% CIs, stratified by type of intervention and timing of diagnosis. Fig. S3. Meta‐analysis of the proportion of patients requiring further endoscopic intervention following IUI. Study‐specific proportions and 95% CIs are shown alongside pooled estimates under common‐effect and random‐effects models. Fig. S4. Pooled proportion of patients requiring additional reconstructive surgery after ureteric injury. Individual study estimates with 95% CIs are reported together with overall pooled effects (common‐effect and random‐effects models). Table S1. Overview of management strategies and corresponding clinical success rates across studies included in the systematic review. [file BJU-138-45-s003.docx]

**Table of contents**

**sFigure 1.** Domain-level risk-of-bias assessment for all included observational studies using the ROBINS-I framework. Each row represents a study and each column corresponds to one of the seven ROBINS-I domains (D1–D7).

**sFigure 2.** Pooled proportion of renal unit loss across included studies (common-effect and random-effects models). Individual study estimates are displayed with 95% confidence intervals, stratified by type of intervention and timing of diagnosis.

**sFigure 3.** Meta-analysis of the proportion of patients requiring further endoscopic intervention following iatrogenic ureteral injury. Study-specific proportions and 95% confidence intervals are shown alongside pooled estimates under common-effect and random-effects models.

**sFigure 4.** Pooled proportion of patients requiring additional reconstructive surgery after ureteral injury. Individual study estimates with 95% confidence intervals are reported together with overall pooled effects (common-effect and random-effects models).

**sTable 1.** Overview of management strategies and corresponding clinical success rates across studies included in the systematic review.

**sFigure 1.** Domain-level risk-of-bias assessment for all included observational studies using the ROBINS-I framework. Each row represents a study and each column corresponds to one of the seven ROBINS-I domains (D1–D7).


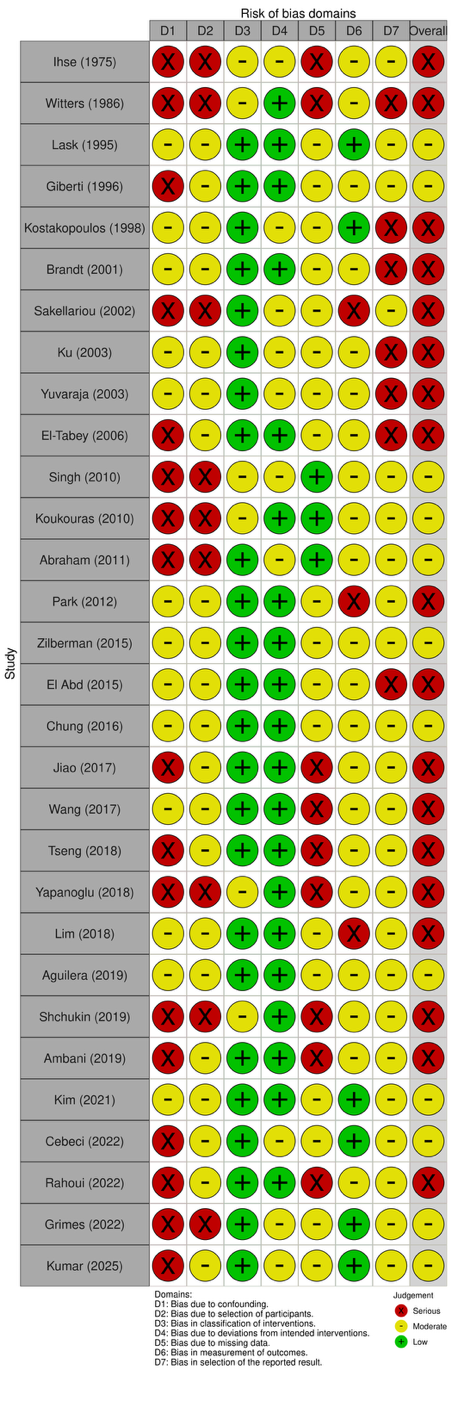


**sFigure 2.** Pooled proportion of renal unit loss across included studies (common-effect and random-effects models). Individual study estimates are displayed with 95% confidence intervals, stratified by type of intervention and timing of diagnosis.

**sFigure 3.** Meta-analysis of the proportion of patients requiring further endoscopic intervention following iatrogenic ureteral injury. Study-specific proportions and 95% confidence intervals are shown alongside pooled estimates under common-effect and random-effects models.

**sFigure 4.** Pooled proportion of patients requiring additional reconstructive surgery after ureteral injury. Individual study estimates with 95% confidence intervals are reported together with overall pooled effects (common-effect and random-effects models).

**sTable 1.** Overview of management strategies and corresponding clinical success rates across studies included in the systematic review.

| Study | Follow-up | Time to recognition | Type of primary management | Clinical success (%) | Clinical success definition | Subsequent reconstructive surgery | Long-term stent dependence | Loss of renal unit |
| --- | --- | --- | --- | --- | --- | --- | --- | --- |
| Ihse (1975) [6] | 0.5–10 y | Mostly intra-op or within first months; strictures sometimes >1 y | Open repairs (mainly UNC, end-to-end); few diversions / nephrectomy  (89% reconstructive) | Overall: 71.4% (30/42) | Symptom-free, acceptable IVP, no further surgery, renal unit preserved | 8/42 pts (19%) re-operated (mainly after E–E) | 0% | 4/42 kidneys (9.5%) nephrectomy |
| Witters (1986) [22] | 1 y | 6 immediate; 20 delayed (most 1–2 wks; 1 at 2 mo) | Immediate/ delayed open repair (UNC, E–E, Boari) ± nephrostomy (6 pts) (89% reconstructive) | Overall: 82% (22/28)  Upfront reconstruction: 100% (20/20) | Symptom resolution + normal IVP + no further intervention | 1 reintervention after UNC/Boari; most nephrostomies needed delayed surgery | 0% | 0% |
| Lask (1995) [7] | 3–6 mo IVP; longer NR | 5–25 d postop (fistulas earlier than cutaneous leaks) | Open surgery (E–E, UNC) or nephrostomy alone  (55% reconstructive) | Overall: 40/44 (91%)  Reconstructive: 24/24 (100%)  Endoscopic: 16/20 (80%) | Normal IVP + no further surgery | 4/20 after nephrostomy needed surgery; none after initial surgery | 0% | 0% |
| Giberti (1996) [8] | 1–12 y | Delayed 2–120 d (mean 20 d) | Immediate or delayed open repair (mainly UNC/UU) and endourology (balloon dilation + stenting )  (60% reconstructive) | Overall: (56/63) 89%  Reconstructive:  (34/38) 89%  Endourology (22/25) 88% | Healed ureter on IVP, no fistula/obstruction, no further surgery | Overall: 6/63 (9.5%);  Reconstructive: 3/38 (7.9%)  Endourology: 3/25 (12%) required | 0% | 2 nephrectomies (irradiated, non-functioning kidneys) |
| Kostakopoulos (1998) [9] | 2–8 y | Intra-op 8 pts; others within 3 wks (leaks) or 3 mo (obstruction) | Reimplantations (immediate or delayed); endoscopic (balloon dilation + stenting )  (65% reconstructive) | Overall: 39/40 = 97.5%;  Endoscopic: 15/15 = 100%;  Mixed:  2/2 = 100% | Radiographic healing without stenosis/obstruction | 1 ureter → nephrectomy after failed repair; some reflux but no surgery | 0% | 1 nephrectomy (persistent stenosis) |
| Brandt (2001) [10] | 2-19 y | 5/47 Immediate repair; 42 delayed repair | Immediate UNC/UU; delayed stent/PCN or open repair  (100% reconstructive) | Overall: 47/47 = 100%; | Radiologic resolution + preserved renal function + no further surgery | 0% | 0% | 1 nephrectomy (ischemic kidney, delayed case) |
| Sakellariou (2002) [15] | Median: 2, range: 2–12 y | 29 intra-op; 47 delayed (days–weeks) | Early: suture, E–E, reimplantation; delayed: catheterization ± ureterolysis/reimplantation;  Endoscopic (stenting)  (72% reconstructive) | Overall: 100%  Reconstructive: 64/64 = 100%  Endoscopic: 12/12 = 100% | Restored ureteral patency, no obstruction/fistula on long-term follow-up | 0% (84% of cases with management with stenting were not successfull) | 0% | 0% |
| Ku (2003) [23] | 2–9 mo urography | Median 52 d (2–382); later in operative-repair group | Open repair (UU/UNC/TUU) vs minimally invasive (stent/PCN)  43% (reconstructive) | Overall (24/30) 80%  Open 13/13 (100%); minimally invasive (11/17) 65%. | Improved/normal urography + symptom resolution + no extra surgery | 6/17 minimally invasive → secondary open repair; none after primary open repair | 0% | 0% |
| Yuvaraja (2003) [19] | 2–8 y (mean 5 y) | 25 intraoperative recognition and 11 postoperative  (Average: 1-9.4 days) | DJ stent or PCN (14), immediate reconstruction (21)  (80% reconstructive) | Overall: 27/34 (79%)  Reconstructive: 20/21 (95%); Endoscopic: 7/10 (70%);  PCN index:  0/4 (0%) | Resolution of obstruction/fistula + preserved function | 1/34 patient require balloon dilatation in the delayed reconstructive | 0% | 1 nephrectomy (late-detected non-functioning kidney) |
| El-Tabey (2006) [24] | Mean 16 ± 5.2 mo | UVF ≤3 mo or >3 mo; obstruction early (mean 14 d); bilateral ligation immediate | PCN ± delayed UNC; UNC/Boari for chronic UVF and UUF; repair of obstructions, bilateral ligations  (100% reconstructive) | Overall: 120/120 (100%) | Normal morphology/function on IVU + no further surgery | All reconstructive procedures were planned; no failures reported | 0% | 0% |
| Singh (2010) [25] | Mean 48.3 mo | UVF/strictures 1–4 wks; bilateral ligation within 1 wk | PCN + delayed UNC/Boari, or immediate UNC; some DJ stents  (83% reconstructive) | Overall: 23/24 patients (96%)  Endoscopic: 3/4 (75%)  Reconstructive: 20/20 (100%) | Symptom resolution + normal IVU + no further reconstruction | Planned staged reconstructions only; no failures of primary repair | 0% | 0% |
| Koukouras (2010) [4] | Mean 13.1 mo | Mean 9.2 d (1–27 d) | Percutaneous minimally invasive: PCN + antegrade recanalization + balloon + int–ext stent  (0% reconstructive) | Overall: 17/24 (72%) patients | Patent ureter on imaging + symptom-free + no open repair | 7/25 ureters → secondary open/laparoscopic repair | 0% | 0% |
| Abraham (2011) [20] | Mean 16.3 mo | Mean 14 d from injury to reconstruction (3 d–2 mo) | Pure laparoscopic reconstructions (mainly UNC ± psoas)  (100% reconstructive) | Overall: 41/41 (100%) patients | Asymptomatic, stable/improved renal function, unobstructed imaging | 0% reoperations | 0% | 0% |
| Park (2012) [1] | 13–15.5 mo | 20 intra-op; 15 delayed 1–28 d | Intra-op: UU, primary repair, UNC; delayed: stent/PCN ± UU/UNC/balloon  (43% reconstructive) | Overall: 27/35 (77%)  Endoscopic: 7/15 (46.7%); Reconstructive: 20/20 (100%) | Normal patency on IVP/CT + symptom resolution + no major re-do surgery | 2 patients needed later ballooning/dilatation | 0% | 0% |
| Zilberman (2015) [21] | 8–24+ mo  (13 pts >2 anni) | Median 9 d (IQR 4–17) | Renal drainage only: nephroureteral stent/stent or nephrostomy  (0% reconstructive) | Overall: 18/29 (62%) | Complete healing on pyelography, no obstruction/leak, no surgery | 6/29 required definitive surgery | 2/29 (6.9%) | 0% |
| El Abd (2015) [11] | Median 28 mo | 27.6% intra-op; 72.4% delayed (days–weeks) | Immediate UNC/UU/Boari vs delayed UNC/reimplantation or endoscopy  (73% reconstructive) | Overall: 79/98 (81%)  Endoscopica: 20/26 (77%)  Reconstructive: 59/72 (82%) | Resolution of obstruction/fistula + patent ureter + no redo | 10/98 reoperations  (all delayed group) | 0% | 0% |
| Chung (2016) [17] | Median 9.7 mo | Mean 17.4 d (0–68 d); 4 suspected intra-op | Retrograde stenting for all; failures → nephrostomy+surgery  (16% reconstructive) | Overall: 16/25 (64%)  Endoscopic: 12/21 (57%) complete healing after stent  Reconstructive: 4/4 (100%) | Healing on CT without stricture requiring surgery | 3/21 required later reconstruction for stricture | NR | NR |
| Jiao (2017) [14] | Mean 11.4 mo | 0–34 d (mean 7.9 d) | CBCT-guided PCN with internal–external or external drainage; some balloon dilations  (0% reconstructive) | Overall: 29/40 (73%) | Catheter removal without surgery, no leak/obstruction on imaging | 5/40 (13%) reconstructive surgery  5/40 (13%) endourologic surgery | NR | 0% |
| Wang (2017) [26] | Mean 36.5 mo | All postoperative after gyn surgery (exact timing NR) | Laparoscopic UU or UNC  (100% reconstructive) | Overall: 56/60 (93%) | No recurrent obstruction/fistula; no re-reconstruction | Further reconstructive/endoscopic: 4/60 (6.7%) | 0% | 0% |
| Tseng (2018) [12] | Median 41.4 mo | NR | Open reconstruction (mainly UNC ± psoas/Boari and UU)  (100% reconstructive) | Overall: 71.4% trifecta (50/70) | No function loss, no hydronephrosis progression, no long-term stent/redo | NR (failures had redo or chronic stent) | 15.7% (11/70) long-term double J | 0% |
| Yapanoglu (2018) [13] | ~1 y | All delayed presentations | Deligation or nephrectomy for ligation; E–E for complete cut; repair + JJ for partial cut; UNC/Boari for UVF  (70% reconstructive) | Overall: 28/30 (93.3%)  Endoscopic/stent-based: 9/9 (100%)  Reconstructive: 19/21 (90%) | No complications at 1-year follow-up. | 0% reoperations | 0% chronic stent | 2 nephrectomies (late untreated ligation) |
| Lim (2018) [18] | Median 13 mo | Strictures after primary UUS; timing not central | Balloon dilation with 12-wk double J after prior UUS  (0% reconstructive) | Overall: 60/102 (61%) | No further intervention ≥12 mo (no chronic stent or re-reconstruction) | 6/102 (5.9%) reconstruction;  24/102 (24%) endoscopic (second balloon dilatation)  12/102 (12%) chronic stent | 14% (14/102) chronic stent | 0% |
| Aguilera (2019) [2] | Mean: 24 mo. | Immediate: 35  Delayed: 49 | Primary reconstructive:  78/84 = 93%  Endoscopic: 6/84 (7.1%)  (93% reconstructive) | Overall: 60/84 (71%)  Reconstructive only:  56/76 = 74% | Creatinine, renal atrophy, vesico-ureteral reflux or ureteral stenosis at CT | 0% | 0% | 2/84 (2.4%) |
| Shchukin (2019) [27] | Mean: 24.6 mo | Mean time: 6.8 ± 2.6 days; 8% intraoperative | Bridge stent or nephrostomy: 31/73 (42%)  Primary reconstruction (Boari flap): 70/73 (96%)  (96% reconstructive) | Overall: 67/73 (92%)  Endoscopic: 3/3 (100%)  Reconstructive: 64/70 (91%) | No symptoms, no obstruction, preserved or mild deterioration of renal function. | 3 Boari flap re-operations, success in 2/3 (66.7%) | 0% | 2/73 (2.7%) |
| Ambani (2019) [28] | Radiologic 15 mo; clinical 22–27 mo | Early group intra-op; delayed group repaired at mean 9.2 mo | All UNC ± psoas/Boari  (100% reconstructive) | Overall: 65/67 (97%) | No recurrent obstruction or stricture on imaging | 1/67 (1.5%) redo UNC + Boari; 1/67 (1.5%) managed with chronic stent | 1/67 chronic stent | 0% |
| Kim (2021) [31] | Median 24.5 mo | 31% <24 h; 69% >24 h | All laparoscopic reconstructions (LEEU or LUN ± psoas)  (100% reconstructive) | Overall: 95.1% (58/61) | Patent ureter on imaging + no further procedures + symptom-free | 3/61 (4.9%) failures managed with balloon + stent  No further reconstruction. | 0% | 0% |
| Cebeci (2022) [16] | Median 58 mo | 48% immediate; 52% delayed (median 12 d) | Endourology (DJ) vs open UNC/UU  (41% reconstructive) | Overall: 19/27 (70%) Endoscopic: 8/16 (50%)  Reconstructive: 11/11 (100%) | Stricture-free ureter + no symptoms + no repeat surgery | Further reconstructive: 5/27 (19%) | 3/27 (11%) | 0% |
| Rahoui (2022) [29] | Mean 36.2 mo | Mean 4.2 d (1–14 d) | Initial stent or PCN; definitive UNC (most) or ileal ureter  (84% reconstructive) | Overall: 26/32 (81%)  Reconstructive: 24/27 (89%)  Endoscopic: 1/5 (20%) | No recurrence/stricture or secondary hydronephrosis | NR | 4/32 (13%) | 9.4% nephrectomy |
| Grimes (2022) [30] | Median 30 mo | Mix of immediate and delayed; most with PCN before reconstruction | Definitive reconstructive surgery (reimplant ± psoas/Boari; ileal ureter; autotransplant)  (100% reconstructive) | Overall: 46/47 (98%) | No need for revision or chronic drainage | 1 revision for early leak | 0% | 0% |
| Kumar (2025) [5] | Up to 2 y | Early <4 wks vs late >4 wks (timing strongly predictive) | Retrograde DJ stenting only; stent 6 wks then remove  (0% reconstructive) | Overall: 77% (24/31) | Fistula closed on CT urography + no leak after removal + no further treatment | 1/31 (3.2%) ureteric reimplantation (retained stent, chronic fistula) | 0% | 0% |

Abbreviations: CBCT = cone-beam CT; CT = computed tomography; DJ = double-J stent; JJ = double-J stent; E–E = end-to-end ureteroureterostomy; IR = interventional radiology; IVP = intravenous pyelogram; IVU = intravenous urography; LEEU = laparoscopic end-to-end ureteroureterostomy; LUN = laparoscopic ureteroneocystostomy; NUS = nephro-ureteral stent; NR = not reported; PCN = percutaneous nephrostomy; TTD = time to diagnosis; TUU = transureteroureterostomy; UU = ureteroureterostomy; UUF = uretero-uterine fistula; UUS = ureteroureterostomy surgery; UVF = ureterovaginal fistula; d = days; wks = weeks; mo = months; y = years; pts = patients.
