# Supplementary material for: Iatrogenic ureteric injuries after abdominal surgery: a systematic review and meta‐regression from the European Association of Urology Endourology Section
Source: BJU Int. 2026 Apr 23;138(1):45–56. doi: 10.1111/bju.70275 (PMC13244928; doi:10.1111/bju.70275)
Supplement: Supplementary file 3 — File S2. Search strategies. [file BJU-138-45-s002.docx]

**Supplementary File 2**

**PICO:**

Population: Adults with iatrogenic ureteral injury related to abdominopelvic or endoscopic surgery, diagnosed intraoperatively or postoperatively.

Intervention: Minimally invasive/endourological management (ureteral stenting, percutaneous nephrostomy, ureteroscopy-based techniques, balloon dilatation, endoureterotomy).

Comparison: Alternative minimally invasive strategies and/or open/laparoscopic reconstructive surgery, or no further intervention.

Outcomes: Success of urinary drainage, spontaneous resolution without major reconstruction, need for reconstructive surgery, renal function, complications, and long-term ureteral patency.

**PUBMED: 1286**

(

("Ureter"[Mesh] OR ureter*[tiab])

AND

(injur*[tiab] OR trauma*[tiab] OR perforat*[tiab] OR lacerat*[tiab]

OR transect*[tiab] OR avulsion*[tiab] OR fistula*[tiab])

AND

(iatrogen*[tiab] OR "intraoperative complications"[Mesh] OR "postoperative complications"[Mesh]

OR gynecolog*[tiab] OR hysterectom*[tiab] OR "pelvic surgery"[tiab]

OR "abdominopelvic surgery"[tiab] OR colorectal[tiab])

)

AND

(

stent*[tiab] OR "ureteral stent"[tiab] OR "double j"[tiab] OR "JJ stent"[tiab]

OR nephrostom*[tiab] OR "percutaneous nephrostomy"[tiab]

OR ureteroscop*[tiab] OR endourolog*[tiab] OR endoscop*[tiab]

OR "balloon dilat*"[tiab] OR endoureterotom*[tiab]

OR "minimally invasive"[tiab]

)

NOT

(

gunshot[tiab] OR stab[tiab] OR "blunt trauma"[tiab] OR "penetrating trauma"[tiab]

)

**EMBASE: 2959**

('ureter'/exp OR ureter*:ti,ab)

AND

(injur*:ti,ab OR trauma*:ti,ab OR perforat*:ti,ab OR lacerat*:ti,ab

OR transect*:ti,ab OR avulsion*:ti,ab OR fistula*:ti,ab OR disruption*:ti,ab)

AND

(iatrogen*:ti,ab OR 'intraoperative complication'/exp OR 'postoperative complication'/exp

OR gynecolog*:ti,ab OR hysterectom*:ti,ab OR 'pelvic surgery':ti,ab

OR 'abdominopelvic surgery':ti,ab OR colorectal:ti,ab OR 'colorectal surgery':ti,ab)

AND

(stent*:ti,ab OR 'ureteral stent':ti,ab OR 'double j':ti,ab OR 'jj stent':ti,ab

OR nephrostom*:ti,ab OR 'percutaneous nephrostomy':ti,ab

OR ureteroscop*:ti,ab OR endourolog*:ti,ab OR endoscop*:ti,ab

OR 'balloon dilat*':ti,ab OR endoureterotom*:ti,ab OR 'minimally invasive':ti,ab

OR 'ureteral reimplantation':ti,ab OR 'ureteral reconstruction':ti,ab)

NOT

('blunt trauma':ti,ab OR 'penetrating trauma':ti,ab OR gunshot:ti,ab OR stab:ti,ab)

AND [humans]/lim

AND [english]/lim
